# The relation of a cerebrospinal fluid profile associated with Alzheimer’s disease with cognitive function and neuropsychiatric symptoms in sporadic cerebral amyloid angiopathy

**DOI:** 10.1186/s13195-024-01454-3

**Published:** 2024-05-04

**Authors:** Anna M. De Kort, Kanishk Kaushik, H. Bea Kuiperij, Lieke Jäkel, Hao Li, Anil M. Tuladhar, Gisela M. Terwindt, Marieke J. H. Wermer, Jurgen A. H. R. Claassen, Catharina J. M. Klijn, Marcel M. Verbeek, Roy P. C. Kessels, Floris H. B. M. Schreuder

**Affiliations:** 1 Department of Neurology, Radboud University Medical Center, Nijmegen, The Netherlands; 2 Donders Institute for Brain, Cognition and Behaviour, Radboud University, Nijmegen, The Netherlands; 3 Radboud Alzheimer Centre, Radboud University Medical Center, Nijmegen, The Netherlands; 4 Department of Neurology, Leiden University Medical Centre, Leiden, The Netherlands; 5 Department of Neurology, University Medical Center Groningen, Groningen, The Netherlands; 6 Department of Cardiovascular Sciences, University of Leicester, Leicester, United Kingdom; 7 Department of Human Genetics, Radboud University Medical Center, Nijmegen, The Netherlands; 8 Department of Medical Psychology, Radboud University Medical Center, Nijmegen, The Netherlands; 9 Vincent van Gogh Institute for Psychiatry, Venray, The Netherlands

## Abstract

**Background:**

Patients with sporadic cerebral amyloid angiopathy (sCAA) frequently report cognitive or neuropsychiatric symptoms. The aim of this study is to investigate whether in patients with sCAA, cognitive impairment and neuropsychiatric symptoms are associated with a cerebrospinal fluid (CSF) biomarker profile associated with Alzheimer’s disease (AD).

**Methods:**

In this cross-sectional study, we included participants with sCAA and dementia- and stroke-free, age- and sex-matched controls, who underwent a lumbar puncture, brain MRI, cognitive assessments, and self-administered and informant-based-questionnaires on neuropsychiatric symptoms. CSF phosphorylated tau, total tau and Aβ42 levels were used to divide sCAA patients in two groups: CAA with (CAA-AD+) or without a CSF biomarker profile associated with AD (CAA-AD-). Performance on global cognition, specific cognitive domains (episodic memory, working memory, processing speed, verbal fluency, visuoconstruction, and executive functioning), presence and severity of neuropsychiatric symptoms, were compared between groups.

**Results:**

sCAA-AD+ (*n*=31; mean age: 72 ± 6; 42%, 61% female) and sCAA-AD- (*n*=23; 70 ± 5; 42% female) participants did not differ with respect to global cognition or type of affected cognitive domain(s). The number or severity of neuropsychiatric symptoms also did not differ between sCAA-AD+ and sCAA-AD- participants. These results did not change after exclusion of patients without prior ICH.

**Conclusions:**

In participants with sCAA, a CSF biomarker profile associated with AD does not impact global cognition or specific cognitive domains, or the presence of neuropsychiatric symptoms.

**Supplementary Information:**

The online version contains supplementary material available at 10.1186/s13195-024-01454-3.

## Introduction

Sporadic cerebral amyloid angiopathy (sCAA), which is characterized by amyloid β accumulation in the vessel wall of small to medium-sized brain cortical and leptomeningeal vessels, is a frequent pathology in the general older population. Up to 23% of the adults above the age of 55 has moderate-to-severe sCAA, but only a minority develops clinical symptoms [1]. These symptoms are diverse: some present with transient focal neurological episodes, while others may have cognitive impairment or intracerebral haemorrhage (ICH) as the first presentation [2]. The disease course in relation to associated MRI abnormalities can also vary greatly between patients: some may never develop dementia while having extensive cerebral small vessel disease (SVD) on MRI, whereas others have a relatively small SVD burden but may experience relatively rapid cognitive decline. In addition, the affected cognitive domain(s) and the severity of cognitive impairment vary amongst patients with CAA [3, 4]. Finally, large interindividual differences exist in the presence, severity, and nature of neuropsychiatric symptoms [5].

The prevalence of mild cognitive impairment or dementia in sCAA is 50% according to a recent systematic review and meta-analysis [6]. One of the factors that may contribute to cognitive decline in patients with sCAA is concomitant Alzheimer’s disease (AD) pathology. A previous study showed that the presence of objective memory impairment and global cognitive impairment in patients with sCAA and mild cognitive impairment was associated with tau-PET retention, which is a marker for AD pathology [7]. In addition, a large population autopsy study suggested that severe CAA pathology was associated with an increased odds of a clinical diagnosis of AD dementia [8]. Furthermore, in a cohort of cognitively unimpaired individuals and individuals with mild cognitive impairment, it was found that cerebrospinal fluid (CSF) phosphorylated tau (p-tau)/Aβ42 (amyloid β 42) ratios (proxy-markers of AD pathology) were associated with neuropsychiatric symptoms [9].

We hypothesize that concomitant AD contributes to cognition dysfunction and to the presence of neuropsychiatric symptoms in patients with sCAA, irrespective of neuroimaging features. To evaluate AD pathology, we have used a commonly used proxy for in vivo AD pathology [9]: a cerebrospinal fluid (CSF) biomarker profile indicative of amyloid deposition (A), tau accumulation (T), and neurodegeneration (N) [10].

We aim to investigate global cognition, the pattern and degree of impairment of affected cognitive domains, and the number and nature of neuropsychiatric symptoms in participants with sCAA with a CSF profile indicative of concomitant AD (sCAA-AD+) versus participants with sCAA without such profile (sCAA-AD-) [10]. Moreover, we will examine whether prior ICH modifies the association between a CSF profile indicative of concomitant AD and any associations.

## Methods

### Participants

We included participants from two studies, which were largely comparable in study procedures, allowing for pooling of data. Patients with sCAA were from the BIONIC (BIOmarkers for cogNitive Impairment due to Cerebral amyloid angiopathy; www.radboudumc.nl/BCS) and the FOCAS (Follow-up in sporadic CAA Study) studies. The BIONIC study is a cross-sectional cohort study on CSF biomarkers for sCAA situated in the Radboud University Medical Center (RUMC), Nijmegen. Patients with probable sCAA according to the modified Boston criteria (version 1.5) [11] were consecutively recruited from the neurology and geriatrics outpatient clinics from the RUMC between December 2018 and July 2023, and from the FOCAS study, a longitudinal study on the disease course of sCAA with comparable inclusion criteria, situated in the Leiden University Medical Center (LUMC) [12]. The diagnosis was verified by a senior vascular neurologist (FHBMS, CJMK, MJHW). Exclusion criteria were contra-indications for lumbar puncture or MRI, and recent (<3 months) symptomatic stroke. In FOCAS, CSF sampling was not performed in all participants; for the current study we selected participants based on the availibility of CSF. Four individuals from the FOCAS study were ultimately diagnosed with mixed CAA; three individuals had one deep microbleed, one individual had three deep microbleeds (signs of concomitant of deep perforating arteriopathy).

Control participants were recruited in the context of the CAFE (Cerebral Amyloid angiopathy Fluid biomarkers Evaluation) study. They were recruited at the RUMC from partners and family of the patients with sCAA. In addition, we recruited individuals via the Dutch Brain Research Registry [13]. Inclusion criteria were age ≥55 years, a Montreal Cognitive Assessment (MoCA) score >28 or a modified Telephone Interview of Cognitive Status (mTICS) score of ≥35 [14, 15]. Additional exclusion criteria for the controls included self-reported (subjective) cognitive decline, and a history of major brain pathology such as spontaneous parenchymal intracerebral hemorrhage, ischemic stroke, neurodegenerative disease, brain tumors, brain infection or inflammation. The controls were age and- sex-matched to the patients with sCAA from the BIONIC study. For details on the BIONIC/CAFE protocol, see the Methods section in the supplement.

### CSF analysis

All participants underwent a lumbar puncture according to local protocols (see supplement). At both participating hospitals, the CSF was collected in polypropylene tubes, centrifuged, aliquoted, and stored in polypropylene tubes at −80°C.

All CSF analyses were performed at the RUMC. Patient and controls samples were randomly analyzed to avoid bias. CSF Aβ40, Aβ42, tau phosphorylated at threonine 181 and total tau levels were quantified using the Lumipulse chemiluminescent immunoassay (Fujirebio, Ghent, Belgium). The samples were analysed in different batches; however, we adhere to strict guidelines under the ISO15189 guidance to control that inter-assay variation is kept within predefined limits of variation for each assay.

We stratified participants into individuals with a biomarker profile indicative of concomitant AD (sCAA-AD+) and those without a biomarker profile indicative of concomitant AD (sCAA-AD-). A biomarker profile indicative of concomitant AD (A+T+N+ or AD+) was defined as a combination of the following: amyloid deposition (A+; a decreased CSF Aβ42 concentration), tau accumulation (T+: an increased CSF phosphorylated tau concentration), and neurodegeneration (N+: an increased total tau concentration) [10] We used the following predefined local cutoff values: (CSF Aβ42 (A+): <659pg/ml; phosphorylated tau (T+): >64pg/ml; total tau (N+): >400pg/ml).

### Neuropsychological assessment

The following cognitive domains were investigated: *Episodic memory* (Rey Auditory Verbal Learning Test (RAVLT) and recall) [16], *Working memory* (mean of Wechsler’s Digit Span Test forward and backward trials), *Processing speed* (Stroop card I, Stroop card II, Trail Making Test A (TMT-A)), *Verbal Fluency* (one minute animal naming), *Visuoconstruction* (Rey-Osterrieth’s Complex Figure Test – Copy trial), *Executive functioning* (Stroop interference score, TMT interference score).

We calculated the Stroop interference score by dividing the Stroop part III score by part II. We calculated TMT interference score by dividing TMT-B by TMT-A. Global cognition was assessed using the MoCA version 7.1 [17].

We scored education level using seven categories in accordance with the Dutch educational system, (the Verhage Score) [18], which is comparable to the UNESCO international classification of education levels [19].

We converted all raw test scores into Z-scores corrected for age, sex and education level for each participant based on a large normative dataset from the Advanced Neuropsychological Diagnostics Infrastructure (ANDI; www.andinorms.nl). The ANDI database includes data from up to 26,000 healthy individuals from all ages [20, 21]. Next, we averaged Z-scores of cognitive tests that reflected the same cognitive domain to get a composite Z-score per cognitive domain. If one test of a particular domain was missing, the domain score was based on the remaining tests of that domain. To correct for extreme performances, Z-scores <-3 and >3 were converted to -3 and 3, resulting in a scoring range of -3 to 3 (reflecting the full range of severely impaired to extremely superior performances). Furthermore, we defined cognitive impairment on a domain as a composite Z-score of <−1.5 [22]. We defined global cognitive impairment as a MoCA Z-score of <−1.5. Single domain cognitive impairment was defined as a Z-score of <1.5 on a single domain, multi domain cognitive impairment was defined as a Z-score or of <-1.5 on more than one domain.

### Neuropsychiatric assessment

Apathy was assessed using the Dutch version of the self-reported Apathy Starkstein Scale (AS) (performed at RUMC only) and informant version of AS (both RUMC and LUMC) [23]. This questionnaire consists of 14 items with four possible answers ranging from 0 to 3 points, with higher scores indicating more severe apathy (range: 0-42 points). A score of at least 14 points is indicative for the presence of apathy. We considered the informant version more valid, since the presence of (mild) cognitive impairment may influence the validity of self-reported apathy [24]. However, we decided to report both the self-reported and informant AS scale, if available.

Neuropsychiatric symptoms were investigated using the Dutch version of the Neuropsychiatric Inventory Questionnaire (NPI) [25] in participants at the RUMC, and by using the shortened version, the NPI-Questionnaire (NPI-Q) [26] in participants at the LUMC. The NPI is a retrospective caregiver-informant interview with screening questions covering 12 neuropsychiatric symptom domains: delusions, hallucinations, agitation/aggression, dysphoria/depression, anxiety, euphoria/elation, apathy/indifference, disinhibition, irritability/lability, aberrant motor behaviours, nighttime behavioural disturbances, and appetite/eating disturbances. Informants are asked to answer “yes” or “no” in response to each screening question, and to rate the severity of the symptoms present in the last 4 weeks if the answer is “yes” on a three-point scale: (1-mild, 2-moderate, 3-severe). In addition, caregiver burden (6-point scale ranging from “0-not at all” to “5-extreme”) is determined.

The NPI and NPI-Q are highly similar [26]; the main difference is that in the NPI, but not in the NPI-Q, the frequency of a symptom is also rated. To pool the total scores of the questionnaires, we excluded the frequency scale from our analysis. The total NPI and NPI-Q severity score represents the sum of individual symptom scores (presence x severity) and ranges from 0 to 36.

### MRI acquisition

Participants at the RUMC (patients with sCAA and controls) underwent brain MRI on a 3.0 Tesla MRI scan (Siemens Magnetom Prisma, Siemens Healthineers, Erlangen, Germany) using a 32-channel head coil. Participants were examined using a comprehensive protocol (Supplementary Table S1). For the current study, 3D T1-weighted sequence using Magnetization Prepared 2 Rapid Acquisition Gradient Echoes (MP2RAGE), the 3D T2-weighted sequence, the 3D fluid-attenuated inversion recovery (FLAIR), and the 3D multi-echo gradient echo T2*-weighted sequence were analyzed. Magnitude and phase data from the multi-echo gradient sequence was processed to a SWI using the “Contrast-weighted, Laplace-unwrapped, bipolar multi-Echo, ASPIRE-combined, homogeneous, improved Resolution SWI” (CLEAR-SWI) method [27]. Participants at the LUMC were scanned on a 3.0 Tesla MRI scanner (Philips Healthcare, Best, the Netherlands) with a 32-channel head coil. This protocol included SWI, T2 and FLAIR sequences. Further details are described in previous reports [12].

### Cerebral small vessel disease markers

We assessed the number and distribution of cerebral microbleeds (CMBs) [28], presence and extent of cortical superficial siderosis (CSS) [11], presence and extent of enlarged perivascular spaces (EPVS) in the centrum semi-ovale (CSO; using a dichotomized classification: high (≥21 EPVS) or low (≤20 EPVS)) [29] and white matter hyperintensities (WMH) according to the Fazekas Scale [30, 31]. Using these four parameters, we calculated a summary score of SVD markers in sCAA [29], referred to as CAA-related SVD burden score, ranging from 0 to 6 points. Two trained readers rated the different SVD makers on the MRI; in case of disagreement, a senior vascular neurologist (FHBMS) or neuroradiologist was consulted to reach final consensus.

### Hippocampus volumetry

The total intracranial volume and hippocampal volume were estimated using the ‘SAMSEG’ function within Freesurfer (version 7.3.2) [32]. Hippocampus segmentation was manually corrected if needed. To account for head size, we computed the ratio of the bilateral hippocampal volumes to the total intracranial volume: normalized hippocampal volume (nHV) = [(right + left hippocampal volumes)/ intracranial volume]. The nHV is available for the RUMC participants only.

### Data analysis

Data are represented as mean ± standard deviation (SD) or median with interquartile range (IQR). We compared demographic, clinical, radiological, neuropsychological test data, and neuropsychiatric questionnaire data between sCAA-AD+ versus sCAA-AD- participants, and between sCAA-AD- participants and controls. We compared sCAA-AD- patients and controls to study the association of sCAA alone with cognition and neuropsychiatric symptoms. Differences were analyzed with a Student’s t-test or Mann-Whitney U test as appropriate. Depending on group size, differences in proportions were compared by a chi-square test, or a Fisher Exact test. In case of more than two categories in a row, the Fisher-Haller-Freeman test was used to assess differences in proportions.

Since prior symptomatic ICH may influence both cognitive function and neuropsychiatric symptoms, we repeated the analyses after exclusion of sCAA patients with prior ICH.

Since CSF Aβ42 is also decreased in sCAA, and p-tau is the most specific for AD, we also analyzed the effect of the individual CSF biomarkers of AD on cognitive performance and neuropsychiatric symptoms. We used binominal logistic regression analyses to analyze the association of separate CSF AD biomarkers, the p-tau/Aβ42 ratio and nHV; with (I) global cognition (impairment vs normal), (II) episodic memory (impairment vs normal), and (III) neuropsychiatric symptoms (1 or more symptoms vs no symptoms) in participants with sCAA, all adjusted for history of ICH. Odds ratios were standardized, and 95% confidence intervals (CI) were calculated. Episodic memory was analyzed since this domain is considered to be specifically impaired in patients with AD [33]. Because cognitive scores were already adjusted for age, sex, and education level, these variables were not additionally included in the models. In the model for neuropsychiatric symptoms, we adjusted for age [33].

### Ethical statement

We obtained written informed consent from all participants. All participants underwent lumbar puncture in the context of studies on biomarkers for CAA, which were approved by the local medical ethics committee of the RUMC (NL 63298.091.17, NL 62669.091.17) and LUMC (NL63256.058.17).

### Data Availability

Anonymized data not published within this article will be made available by request from any qualified investigator.

## Results

We included 57 participants with sCAA (38 from BIONIC, and 19 from FOCAS) and 28 controls. Overall, 26/57 (45%) participants with sCAA and 4/28 (13%) controls that had a CSF profile indicative of AD (p=0.002). Neuropsychological test data was missing in three participants with sCAA, and neuropsychiatric assessments were missing in three other participants with sCAA. Therefore, the analysis on cognitive profile (54 participants with sCAA and 28 controls) was performed on a slightly different subset of participants compared with the analysis on neuropsychiatric symptoms (54 participants with sCAA and 28 controls; see supplemental methods and results).

### Demographics, small vessel disease markers and neurocognitive profile

Age, sex and level of education were well-balanced between the two groups of participants with sCAA (Tables 1 and 2). The distribution of SVD markers did not differ between CAA-AD+ and CAA-AD- patients. nHV was lower in the sCAA-AD+ participants compared to the sCAA-AD- participants (Table 1). In addition, the nHV of sCAA-AD- participants was also lower compared to controls (*p*=0.018).

**Table 1:** Demographics, clinical characteristics, cardiovascular risk factors, MRI and CSF parameters of participants with sCAA and controls with available neuropsychological examinations

	**sCAA-AD+ (*n*=23)**	**sCAA-AD- (*n*=31)**	**Controls (*n*=28)**	***P*-value sCAA-AD+ vs sCAA AD-**
Demographics				
Age (years) (mean ±SD)	72 ± 6	70 ± 5	71 ± 7	0.35^a^
Female sex (n, (%))	14 (61%)	13 (42%)	13 (46%)	0.17^b^
Education score (median, IQR)	5 [4–6]	6 [5–6]	6 [5–7]	0.39^c^
History (n, (%))				
Prior symptomatic ICH	3 (13%)	11 (36%)	-	0.12^b^
Vascular risk factors (n, (%))				
Hypertension	15 (68%)*	17 (55%)*	14 (50%)	0.40^b^
Hypercholesterolaemia	14 (63%)*	18 (62%)**	9 (33%)*	0.61^b^
Diabetes	2 (9%)	1 (3%)	2 (7%)	1.0^c^
Smoking	20 (74%)	17 (65%)	18 (64%)	0.46^b^
History of ischemic stroke	1 (4%)	4 (13%)	0 (0%)	0.28^c^
History of TIA	2 (9%)	4 (13%)	0 (0%)	1.0^c^
History of cardiovascular disease	2 (9%)	7 (30%)*	3 (11%)	0.18^c^
MRI parameters (n, (%))				
lobar CMB category				
0 or 1 CMBs	1 (4%)	2 (7%)	28 (100%)	0.25^c^
2-4 CMBs	3 (13%)	6 (19%)	0 (0%)	
5-10 CMBs	5 (22%)	6 (19%)	0 (0%)	
11-20 CMBs	1 (4%)	6 (19%)	0 (0%)	
>21 CMBs	13 (57%)	11 (36%)	0 (0%)	
CSS category				0.59^a^
no CSS	7 (30%)	13 (42%)	28 (100%)	
focal CSS	5 (22%)	4 (13%)	0 (0%)	
disseminated CSS	11 (48%)	14 (45%)	0 (0%)	
>20 EPVS-CSO	19 (83%)	30 (97%)	21 (75%)	0.15^d^
Fazekas Score (median, IQR)	3 [2–3]	2 [2–3]	1 [1-1]	0.35^a^
CAA-SVD burden score (median, IQR)	4 [4–6]	4 [3–5]	1 [1-1]	0.39^a^
nHV^e^ (median, IQR)	4.63 [4.71-5.48]	5.15 [4.17-5.48]	5.36 [5.09-5.71]	**0.007**^**a**^
CSF parameters (median, IQR)				
Aβ_42_ (pg/ml)	336 [252-390]	398 [288-562]	1030 [802-1314]	-
Total tau (pg/ml)	600 [501-783]	391 [276-447]	365 [280-508]	-
Phospho-tau (pg/ml)	78 [70-114]	46 [34-57]	42 [34-68]	-

Percentages were rounded so may not always add up to 100%

Abbreviations: *sCAA* sporadic cerebral amyloid angiopathy, *CMB* Cerebral microbleeds, *CSS* Cortical superficial siderosis, *EPVS-CSO* Enlarged perivascular spaces in the centrum semiovale, *IQR* Interquartile range, *nHV* Normalized hippocampal volume, *SVD* Small vessel disease, *TIA* Transient ischemic attack

^*^Status unknown for 1 individual, ** Status unknown for 2 individuals

^a^Mann-Whitney U test

^b^chi-square test

^c^Fisher-Freeman-Halton Exact Test

^d^Fisher exact test

^e^BIONIC only

**Table 2:** Neuropsychological scores and percentage of patients with cognitive impairment on a cognitive domain of participants with sCAA stratified to CSF AD biomarker status and controls

	**sCAA-AD+ (*n*=23)**	**sCAA-AD- (*n*=31)**	**Controls (*n*=28)**	***P*-value sCAA-AD+ vs sCAA-AD-**	***P*-value sCAA-AD- vs controls**
MoCA (median raw score, IQR)	26 [20–28]	25 [23–28]	28 [26–29]	0.59^a^	**<0.001**^**a**^
MoCA (median z-score, IQR)	-0.53 [-2.31-0.53]	-0.69 [-1.38-0.34]	0.49 [-0.17-0.93]	0.62^a^	**0.001**^**a**^
Episodic memory (median z-score, IQR)	-1.31* [-2.14 to -0.07]	-0.64 [-1.60-0.33]	0.45 [-0.04-0.98]	0.31^a^	**<0.001**^**a**^
Working memory (median z-score, IQR)	-0.005** [-0.78-0.33]	-0.63 [-0.99-0.39]	0.11 [-0.43-0.63]	0.21^a^	**0.026**^**a**^
Executive function (median z-score, IQR)	-0.25* [-0.77-0.20]	0.43 [-0.39-0.60]	0.71 [0.03-1.11]	0.07^a^	**0.029**^**a**^
Processing speed (median z-score, IQR)	-1.06* [-2.39-0.12]	-1.15 [-1.87 to -0.09]	0.05 [-0.53-0.69]	0.86^a^	**<0.001**^**a**^
Visuospatial^d^ (median z-score, IQR)	-0.54 [-1.25-0.68]	-0.57 [-1.26 to -0.10]	0.85 [0.63-1.49]	0.64^a^	**<0.001**^**a**^
Verbal fluency (median z-score, IQR)	-0.66* [-1.31-1.01]	-0.57 [-1.15-0.13]	0.38 [-0.14-0.76]	0.79^a^	**<0.001**^**a**^
CI per domain (n (%))					
Global cognition	10 (44%)	7 (23%)	0	0.14^b^	**0.011**^**c**^
Episodic memory	7 (32%)*	8 (26%)	0	0.63^b^	**0.005**^**c**^
Working memory	2 (10%)**	3 (10%)	0	1.0^c^	0.24^c^
Executive function	4 (18%)*	3 (10%)	0	0.43^c^	0.24^c^
Processing speed	8 (35%)*	11 (36%)	0	0.96^b^	**<0.001**^**c**^
Visuospatial^d^	2 (14%)	4 (17%)	0	1.0^c^	**0.039**^**c**^
Verbal fluency	4 (18%)*	6 (19%)	0	1.0^c^	**0.025**^**c**^
Single domain CI^e^	3 (14%)*	6 (19%)	0	0.72^c^	**0.025**^**c**^
Multidomain CI^e^	8 (37%)*	10 (33%)	0	0.76^b^	**<0.001**^**c**^

Percentages are rounded so may not be always add up to 100%

Abbreviations: *AD* Alzheimer’s disease, *sCAA* sporadic cerebral amyloid angiopathy, *CI* Cognitive impairment (Z-score below -1.5), *IQR* Interquartile range, *MoCA* Montreal Cognitive Assessment

^*^Data missing from 1 individual

^**^Data missing from 2 individuals

^a^Mann-Whitney U test

^b^chi-square test

^c^Fisher exact test

^d^BIONIC only; 14 sCAA with AD pathology, 24 sCAA patients without AD pathology

^e^the visuospatial domain is missing in patients from LUMC, multidomain cognitive impairment is based on one domain less than in RUMC assessments

MoCA Z-scores were similar between sCAA participants with and without a CSF profile indicative of AD (Table 2). Furthermore, there were no differences in median Z-scores or proportions of participants with impaired global cognition, cognitive impairment on a specific cognitive domain, or in proportions of participants with single or multidomain cognitive impairment between sCAA-AD+ versus sCAA-AD- participants. sCAA-AD- participants had a significantly lower Z-score on global cognition compared to controls and on all other cognitive domains (Table 2). In addition, sCAA-AD- participants had a higher proportion of individuals with impaired global cognition, episodic memory, processing speed, visuoconstruction, verbal fluency, and single domain- and multidomain cognitive impairment compared to controls (Table 2).

### Subgroup analysis excluding sCAA participants with prior ICH

After exclusion of sCAA participants with a prior ICH (*n*=14), we found that sCAA-AD+ (*n*=20) and sCAA-AD- participants (*n*=20) did not differ regarding SVD marker distribution (Table S2). In addition, the nHV was smaller in the sCAA-AD+ participants compared to sCAA-AD- participants (4.59 [4.37-4.93] vs 5.19 [4.87-5.51]; *p*=0.004). MoCA Z-score did not differ between sCAA-AD+ and sCAA-AD- participants (Table S2). Furthermore, there were no differences in median Z-scores of specific cognitive domains the sCAA-AD+ participants, compared to the sCAA-AD- participants. Global cognition and all other cognitive domains were not more often impaired in sCAA-AD+ participants compared to the sCAA-AD- participants.

### Neuropsychiatric symptoms

Presence of apathy, and apathy scores did not differ between sCAA-AD+ and sCAA-AD- participants (Table 3). There were no significant differences regarding the individually reported NPI-Q symptoms and total NPI-Q score between sCAA-AD+ and sCAA-AD- participants. sCAA-AD- participants had more often and more severe apathy compared to controls, based on both the self-reported and informant-based Apathy Starkstein Scale. Based on the NPI-Q, sCAA-AD- participants also had more often symptoms of apathy/indifference and irritability/lability, a higher NPI-Q total score and generally more symptoms than controls (Table 3).

**Table 3:** Scores on neuropsychiatric questionnaires in patients with sCAA, stratified to CSF AD biomarker status and controls

	**sCAA-AD+** **(*n*=26)**	**sCAA-AD-** **(*n*=28)**	**Controls (*n*=28)**	***P*-value sCAA-AD+ vs CAA-AD-**	***P*-value sCAA-AD- vs controls**
Apathy- self reported^e^					
Apathy score (median, IQR)	10 [4–17]	9 [5–17]	6 [4–8]	0.96^a^*	**0.028**^**a**^
Apathy, presence (n, %)	5 (35%)	6 (36%)	1 (4%)	1.0^b^*	**0.007**^**b**^
Apathy- informant					
Apathy score (median, IQR)	12 [6–21]*	12 [8–18]	5 [3–8]	0.73^a^	**<0.001**^**a**^
Apathy presence (n, %)	11 (44%)*	10 (36%)	0	0.54^c^	**<0.001**^**c**^
NPI-Q symptoms (n, %)					
Delusions	1 (4%)	0	0	0.48^c^	-
Hallucinations	0	0	0	-	-
Agitation/aggression	9 (35%)	4 (14%)	0	0.08^c^	0.11^c^
Dysphoria/depression	7 (27%)	3 (11%)	1 (4%)	0.12^c^	0.62^c^
Anxiety	4 (15%)	2 (7%)	0	0.30^c^	0.49^c^
Euphoria/elation	1 (4%)	3 (11%)	0	0.61^c^	0.24^c^
Apathy/indifference	7 (27%)	8 (27%)	0	0.57^c^	**0.004**^**c**^
Disinhibition	2 (8%)	3 (11%)	0	1.0^c^	0.24^c^
Irritability/lability	12 (46%)	7 (25%)	0	0.16^c^	**0.010**^**c**^
Aberrant motor behaviours	0	1 (4%)	0	1.0^c^	1.0^c^
Nighttime behavioural disturbances	3 (12%)	2 (7%)	0	0.66^c^	0.49^c^
Appetite/eating disturbances	4 (15%)	2 (7%)	0	0.41^c^	0.49^c^
NPI-Q Total score (median, IQR)	2 [0-5]	0 [0-3]	0 [0-0]	0.21^a^	**<0.001**^**a**^
NPI-Q no. of symptoms				0.34^d^	**<0.001**^**d**^
No symptoms (n, %)	9 (35%)	15 (54%)	1 (4%)		
1 or 2 symptoms (n, %)	7 (7%)	3 (11%)	0		
3 or more symptoms (n, %)	10 (38%)	5 (18%)	0		

Percentages are rounded so may not be always add up to 100%

Abbreviations: *CAA* Cerebral amyloid angiopathy, *CSF* Cerebrospinal fluid, *NPI-Q* Neuropsychiatric inventory questionnaire, *IQR* Interquartile range

^*^Data missing from 1 individual

^a^Mann-Whitney U test

^b^chi-square Exact Test

^c^Fisher exact test chi-square test

^d^Fisher-Freeman-Halton Exact Test

^e^BIONIC only; 14 sCAA with AD pathology, 20 sCAA patients without AD pathology, 28 controls

### Subgroup analysis excluding sCAA participants with prior ICH

The subgroup analysis excluding sCAA participants with prior ICH showed neither significant differences regarding apathy prevalence or apathy scores, nor in the number and pattern of neuropsychiatric symptoms between sCAA-AD+ and sCAA-AD- participants (Table S3).

### Associations of individual CSF markers, nHV and history of ICH with impairment of global cognition, episodic memory, and presence of neuropsychiatric symptoms in participants with sCAA

**Fig. 1:**
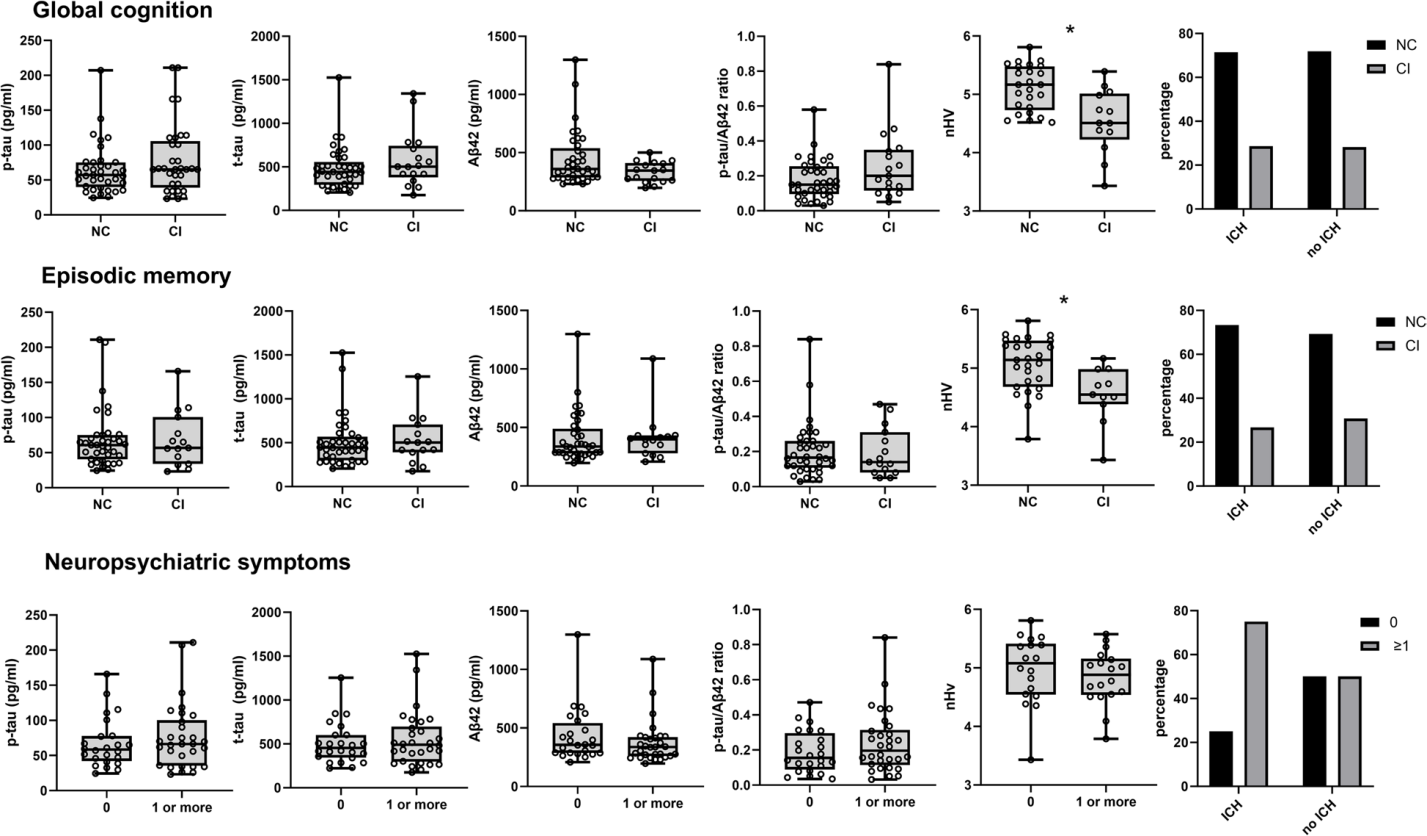
Concentrations of CSF p-tau levels, CSF t-tau levels, CSF Aβ42 levels, CSF p-tau/Aβ42 ratio, nHV, and history of ICH in relation to global cognition (normal vs impairment), episodic memory (normal vs impairment) and psychiatric symptoms (no vs $\ge$ 1 symptoms) in patients with sCAA. Stars (*) indicate a significant odds ratio. Abbreviations**:** CI=cognitive impairment, p-tau= phosphorylated tau, t-tau= total tau, NC=normal cognition, nHV=normalized hippocampal volume

nHV (OR: 4.37 (95% CI: 1.49-12.80); *p*=0.007) was significantly associated with impaired global cognition (Table 4; Fig. 1). However, phosphorylated tau, total tau, Aβ42, p-tau/Aβ42 ratio, and prior ICH were not associated with impaired global cognition. Furthermore, nHV was associated with episodic memory impairment (OR: 3.62 (95% CI: (1.30-10.10); p=0.014), but none of the CSF AD biomarkers nor history of ICH had such association.

**Table 4:** Standardized odds ratios of p-tau, t-tau, Aβ42, p-tau/Aβ42, nHV, and prior ICH in relation to global cognition (normal vs impairment), episodic memory (normal vs impairment) and psychiatric symptoms (no vs $\ge$ 1) in patients with sCAA

**Global cognition (impairment vs normal; corrected for ICH; *n*=54)**		
	**Standardized odds ratio (95% CI)**	***p*-value**
p-tau	0.79 (0.44-1.40)	0.42
t-tau	0.74 (0.42-1.32)	0.31
Aβ42	2.71 (0.87-8.45)	0.09
p-tau/Aβ42 ratio	0.61 (0.32-1.15)	0.13
nHV^**b**^	4.37 (1.49-12.80)	**0.007**
ICH^a^	1.97 (0.47-8.27)	0.35
**Episodic memory (impairment vs normal; corrected for ICH; *n*=53)**		
p-tau	0.99 (0.53-1.82)	0.97
t-tau	0.90 (0.50-1.63)	0.74
Aβ42	0.98 (0.53-1.83)	0.95
p-tau/ Aβ42 ratio	1.05 (0.57-1.97)	0.89
nHV^**b**^	3.62 (1.30-10.10)	**0.014**
ICH^**a**^	0.98 (0.25-3.80)	0.98
**Psychiatric symptoms (** $\ge$** 1 vs 0 symptom(s); corrected for ICH; *n*=54)**		
p-tau	0.69 (0.38-1.27)	0.23
t-tau	0.75 (0.41-1.35)	0.33
Aβ42	1.92 (0.95-3.88)	0.07
p-tau/ Aβ42 ratio	0.55 (0.27-1.09)	0.08
nHV^**b**^	1.34 (0.67-2.66)	0.40
ICH^**a**^	0.33 (0.08-1.41)	0.14

^a^Not corrected for ICH, ^b^BIONIC only; n = 38 sCAA. Aβ42 = CSF amyloid beta-42. *CI* Confidence interval, *CSF* Cerebrospinal fluid, *ICH* Intracerebral haemorrhage, *p-tau* CSF phosphorylated tau, *t-tau* CSF total tau, *nHV* normalized hippocampal volume

There were no factors associated with presence of neuropsychiatric symptoms.

## Discussion

The main findings of our study are as follows: 1) a CSF profile indicative of AD in participants with sCAA is neither associated with impaired global cognition, nor with a specific profile of cognitive impairment, 2) a CSF profile indicative of AD in participants with sCAA was associated with a lower hippocampal volume, but not with white matter hyperintensities or other markers of small vessel disease, 3) a CSF profile indicative of AD in participants with sCAA was neither associated with specific neuropsychiatric symptoms nor with prevalence of neuropsychiatric symptoms.

A clinical study among 63 patients with CAA found a prevalence of CSF AD co-pathology of 50% [34]. Despite that concomitant AD was defined slightly different (a decreased CSF Aβ42, and either an increased phosphorylated tau or total tau, or both) compared to our study, we found a similar prevalence (45%). We hypothesized that concomitant AD in sCAA is associated with a worse cognition, and especially with a worse performance on the episodic memory domain. However, we found that, although there was a trend towards a higher proportion of impaired global cognition in sCAA patients with a CSF profile indicative of AD pathology, the pattern and severity of impairment on specific cognitive domains did not differ between sCAA patients with and without a CSF profile indicative of AD pathology. This may be explained by the fact that the group of sCAA patients we have included was rather heterogeneous, which may hamper detecting an effect. In our sample, the cognition of participants ranged from normal to dementia, and we also included patients with ICH, which can affect cognition as well. A study that included a less heterogenous group of patients (*n*=40), i.e. patients with probable CAA with mild cognitive impairment without prior ICH, found that CAA patients with elevated tau-PET retention as a marker for AD pathology, performed worse in the memory domain and had overall worse cognition (as measured with the Mini-Mental State Examination; MMSE) [7]. On the other hand, a study in 63 CAA participants with a more diverse clinical presentation found similar MMSE scores between CAA patients with and without a CSF profile indicative of AD [34]. Thus, the association between AD co-pathology with a cognitive impairment or impairment on a specific domain might only be detected in a relatively homogeneous group of patients, in patients without prior ICH, or in a specific stage of cognitive impairment. We therefore appreciate the effect of AD co-pathology on the global cognition in the patients with sCAA to be relatively modest.

Alternatively, a CSF profile indicative of AD pathology in sCAA patients might be associated with accelerated cognitive deterioration over time, rather than differences observed in a cross-sectional study design. Based on our cross-sectional data, we cannot exclude an association of a CSF profile indicative of AD pathology with cognitive decline over time. Other studies have demonstrated that a CSF profile indicative of AD pathology was associated with accelerated cognitive decline in memory clinic patients [35], in individuals with subjective memory decline or MCI [36, 37] and in cognitively unimpaired individuals [38].

In the AD continuum, hippocampal atrophy is generally argued to be a phenomenon that occurs later than abnormal CSF biomarkers [39]. Thus, another explanation may be that hippocampal atrophy is a more accurate biomarker for AD pathology than abnormal CSF AD biomarkers, especially since hippocampal volume correlates with global cognition and episodic memory. However, CAA may also affect hippocampal atrophy. This is supported by the fact that we also found a decreased hippocampal volume in CAA patients *without* a CSF profile indicative of AD compared to controls. Another study in patients with sCAA also found a significantly smaller left hippocampal volume compared to controls, especially in patients with cortical superficial siderosis [40]. However, this study did not examine a specific marker for AD pathology [40]. It has been proposed that association between hippocampal atrophy and CAA may be explained by hippocampal hypoperfusion due to reduced cerebral blood flow in the temporal and parietal lobes in patients with CAA [40, 41].

Our results indicate that CSF AD biomarkers in patients with sCAA should be interpreted with caution. When patients are cognitively unimpaired, the clinical relevance of a CSF profile indicative of AD pathology is unclear since there are no data yet on the possible long-term consequences in sCAA patients. When patients are cognitively impaired, it is still not possible to distinguish what the contribution of either CAA or AD co-pathology is to the impairments in specific cognitive domains, based on our data. What we do know, is that sCAA can lead to memory impairment in the absence of AD pathology, since we found that sCAA patients without a CSF profile indicative of AD also had a significantly lower median Z-score on the episodic memory domain score compared to controls. In addition, a longitudinal clinical-pathologic study found that CAA pathology was associated with (amongst others) a decline in episodic memory and cognition [42]. Earlier studies have also demonstrated that vascular dysfunction in sCAA disrupts brain networks, which in turn is associated with atrophy and cognitive impairment [43, 44].

A large autopsy study (*n*=1808) found that CAA pathology was associated with delusions, hallucinations, agitation/aggression, anxiety, apathy, irritability/lability, aberrant motor behavior, and nighttime behavior, as measured with the NPI-Q [45]. This was largely similar to the pattern of neuropsychiatric symptoms associated with AD pathology. This may explain why we found no differences between prevalence of specific psychiatric symptoms, numbers of psychiatric symptoms or total NPI-Q score between sCAA patients with and without a CSF profile indicative of AD, and neither an association of individual CSF AD markers and the presence of neuropsychiatric symptoms. Furthermore, we found that sCAA participants without a CSF profile indicative of AD also had more often apathy and a higher number of neuropsychiatric compared to controls. In addition, a previous study among ICH survivors found that a sCAA MRI profile was associated with severity of neuropsychiatric symptoms [46]. This, together with our findings, indicates that clinicians should be extra alert on neuropsychiatric symptoms in patients with sCAA.

A strength of our study is our design: of a thoroughly characterized cohort with detailed information on cognition, neuro-imaging markers and CSF biomarkers. A limitation of this study is that we used CSF Aβ42 (next to phosphorylated tau and total tau) to define AD pathology, while Aβ42 is also decreased as a result of CAA pathology [47]. However, compared to Aβ42, phosphorylated tau is more specific for AD pathology. Another limitation of our study is the relatively small sample size which may have led to a limited power to detect the effects of a CSF profile indicative of AD. Moreover, the inclusion of participants may be biased to less severely affected patients due to the extensive study protocol. In addition, we did not correct for multiple comparisons in this small, explorative study. Furthermore, the cross-sectional design of our study hampers the evaluation of longitudinal associations between AD pathology and the occurrence of cognitive deterioration or neuropsychiatric symptoms. This should be investigated in future studies.

## Conclusion

In participants with sCAA, a CSF biomarker profile associated with AD does not impact global cognition or specific cognitive domains, or the presence or severity of neuropsychiatric symptoms.

### Supplementary Information

**Supplementary Material File 1.**

## Data Availability

No datasets were generated or analysed during the current study.
