# Supplementary material for: The relation of a cerebrospinal fluid profile associated with Alzheimer’s disease with cognitive function and neuropsychiatric symptoms in sporadic cerebral amyloid angiopathy
Source: Alzheimers Res Ther. 2024 May 4;16:99. doi: 10.1186/s13195-024-01454-3 (PMC11069247; doi:10.1186/s13195-024-01454-3)
Supplement: Supplementary file 1 — Supplementary Material File 1. [file 13195_2024_1454_MOESM1_ESM.docx]

**Supplement to:
The relation of a cerebrospinal fluid profile indicative of Alzheimer’s disease with cognition and neuropsychiatric symptoms in patients with sporadic cerebral amyloid angiopathy**

Anna M. De Kort^1,2,3^, Kanishk Kaushik^4^, H. Bea Kuiperij^1,2,3^, Lieke Jäkel^1,2,3^, Hao Li^1,2^, Anil M. Tuladhar^1,2^, Gisela M. Terwindt^4^, Marieke J.H. Wermer^4,5^, Jurgen A.H.R. Claassen^2,3,6^, Catharina J.M*.*Klijn^1,2^, Marcel M. Verbeek^1,2,3,7^, Roy P.C. Kessels^2,3,8,9^ , Floris H.B.M. Schreuder^1,2

1^Department of Neurology, Radboud University Medical Center, Nijmegen, the Netherlands
^2^Donders Institute for Brain, Cognition and Behaviour, Radboud University, Nijmegen, The Netherlands
^3^Radboud Alzheimer Centre, Radboud University Medical Center, Nijmegen, The Netherlands

^4^Department of Neurology, Leiden University Medical Centre, Leiden, The Netherlands

^5^Department of Neurology, University Medical Center Groningen, Groningen, the Netherlands

^6^Department of Cardiovascular Sciences, University of Leicester, United Kingdom

^7^Department of Human Genetics, Radboud University Medical Center, Nijmegen, The Netherlands

^8^Department of Medical Psychology, Radboud University Medical Center, Nijmegen, The Netherlands

^9^Vincent van Gogh Institute for Psychiatry, Venray, The Netherlands

**Correspondence to:** Floris Schreuder, Department of Neurology, Radboudumc, The Netherlands. Email: floris.schreuder@radboudumc.nl

**Index**Methods
 The BIONIC/CAFE study protocol
 Table S1: MRI acquisition details
Results
 Excluded and incomplete data regarding neuropsychological tests
 Excluded and incomplete data regarding neuropsychiatric questionnaires
 Table S2: Demographics, MRI parameters, neuropsychological scores, and percentage of cognitive impairment on a specific cognitive domain in participants with sCAA without prior ICH, stratified to CSF profile indicative of AD
 Table S3: Scores on neuropsychiatric questionnaires in participants with sCAA without prior ICH, stratified to CSF profile indicative of AD

**Methods**

The BIONIC/CAFE study protocol (relevant to this study) is described below.

- *Medical history*
  With the aid of a structured, standardized questionnaire, each participant was asked about medical history and current medication use. This was subsequently verified with available medical documentation.
  - Hypertension was defined as the use of an antihypertensive drug, or a mean blood pressure >140/90 mmHg during the 30-minute measurement.
  - Hypercholesteremia was defined as a total cholesterol >6.2 mmol/l or using lipid-lowering drugs.
  - Diabetes was defined as the presence of diabetes type 1 or type 2 in medical history.
  - Smoking was defined as a past and/or current smoking habit.
- *Physical assessment*
  Blood pressure and pulse rate were measured in sitting position and was initially assessed on both sides. Then, the blood pressure was measured on the side with the highest blood pressure, every 3 minutes during 30 minutes with a Carescape v100 device, GE Healthcare, Chicago, the Netherlands.
- *Questionnaires to the patient:*
  - Self-reported Starkstein Apathy scale (1)
- *Questionnaires to the informant:*
  - Neuropsychiatric Inventory (2)
  - Starkstein Apathy Scale (1)
- *Neuropsychological examination including the following tests:*
  - MoCA version 7.1 (3)
  - Rey auditory Verbal Learning Test (RAVLT) (4)
  - Trial Making Test A and B (5)
  - Rey-Osterrieth’s Complex Figure Test – Copy trial (6)
  - Symbol Digit Modalities Test (7)
  - Digit Span Task (8)
  - Stroop test (9)
  - One minute Animal naming (10)
- *Lumbar puncture*

Lumbar puncture was performed in left-lateral supine position at the lower lumbar vertebrae using an atraumatic 20-gauge needle (Sprotte, Pajunk, 90-120mm length) after local anesthesia with lidocaine. In case lumbar puncture in supine position was unsuccessful after two attempts, a final attempt in prone position was performed. CSF samples were collected in polypropylene tubes, centrifuged, aliquoted, and stored at −80 °C until analysis.

- *Brain MRI*

A standardized MRI including T1, T2, quantitative susceptibility mapping (QSM), Fluid-attenuated inversion recovery (FLAIR) sequences (Table S1)

**Table S1: Magnetic resonance imaging acquisition details of the BIONIC/CAFE study.**

| **Sequence** | **Parameter** | **RUMC** |
| --- | --- | --- |
| 3D-T1 (MP2RAGE) | TR (ms) | 5000 |
|  | TE (ms) | 3.02 |
|  | TI (ms) | TI1: 732,  TI2: 2500 |
|  | Voxelsize (mm) | 0.8 isotropic |
|  | Flip angle α_1_/α_2_ **(°)** | 4/5 |
|  | Slices | 288 |
|  | Acquisition time | 6:35 |
|  | Multiband acceleration | 8 |
| 3D-T2 | TR (ms) | 3200 ms |
|  | TE (ms) | 563 |
|  | Voxelsize (mm) | 0.8 isotropic |
|  | Slices | 208 |
|  | Acquisition time | 5:57 |
| 3D-FLAIR | TR (ms) | 5000 |
|  | TE (ms) | 394 |
|  | TI (ms) | 1800 |
|  | Voxelsize (mm) | 0.8 isotropic |
|  | Slices | 192 |
| 3D-GRE | Acquisition time | 7:02 |
|  | TE (ms) | TE 1: 6.14 TE 2: 10.14 TE 3: 14.14 TE 4: 18.14 TE 5: 22.14 TE 6: 26.16 TE 7: 30.14  TE 8: 34.14 TE 9: 38.14 |
|  | Voxelsize | 0.8 isotropic |
|  | Flip angle **(°)** | 20 |
|  | Field of view (mm) | 230 |
|  | Acquisition time | 9:25 |
|  | Slices | 176 |

Abbreviations: FLAIR = fluid-attenuated inversion recovery; 3D-GRE = 3D multi-echo gradient echo T2*-weighted sequence; MP2RAGE = magnetization prepared (2) rapid acquisition gradient echoes; mm = millimeter, ms=milliseconds; TE = echo time; TI = inversion time; TR = repetition time.

**Results** *Excluded and incomplete data regarding neuropsychological tests*

Five individuals were colourblind, so Stroop card II and III were not reliable in these patients; their data on these tests was excluded. Two participants with sCAA from RUMC were not able to complete the TMT-B test and Stroop card III because they repeatedly did not understand the instruction; these tests were set at -3 SD. From three participants with sCAA from the LUMC (including two patients with mixed sCAA), either cognitive testing results or education level were unavailable; they were excluded from the analysis. One participant from the LUMC with sCAA had only test data about TMT and the Stroop test. One participant from the LUMC with sCAA had only test data about RAVLT, TMT, Rey figure copy, the Stroop test and Animal fluency. Thus, there were 54 participants with sCAA and 28 controls that had both neuropsychological testing results and CSF available.

*Excluded and incomplete data regarding neuropsychiatric questionnaires*

The AS-informant score is missing in two participants with sCAA from the RUMC and in two participants with sCAA from the LUMC. The self reported apathy score is missing in one control. The NPI is missing in two participants from RUMC and the NPI-Q is missing in one patient from LUMC. Thus, there were 54 patients with sCAA and 28 controls that had both informant-based NPI-(Q) testing results and CSF available.

**Table S2: Demographics, MRI parameters, neuropsychological scores, and percentage of cognitive impairment on a specific cognitive domain in participants with sCAA without prior ICH, stratified to CSF profile indicative of AD**

|  | **sCAA-AD+, no ICH (n=20)** | **sCAA-AD-, no ICH (n=20)** | **P-value CAA AD+ vs sCAA AD-** |
| --- | --- | --- | --- |
| MRI parameters  lobar CMB category  0 or 1 CMBs  2-4 CMBs  5-10 CMBs  11-20 CMBs  >21 CMBs  CSS category  no CSS  focal   disseminated  >20 EPVS-CSO  Fazekas Score  CAA-SVD burden score  nHV | 0 (0%)  2 (10%) 5 (25%) 1 (5%) 12 (60%)  6 (30%) 4 (20%) 10 (50%) 17 (85%) 3 [2-3] 4 [4-6] 4.59 [4.37-4.93] | 2 (10%) 4 (20%) 4 (20%) 5 (25%) 5 (25%)  8 (40%) 3 (15%) 9 (45%) 20 (100%) 2 [2-3] 4 [3-5] 5.19 [4.87-5.51] | 0.08^c^      0.84^c^  0.26^a^ 0.25^a^ 0.27^a^ **0.004^a^** |
| MoCA (median raw score, IQR) | 23 [19-28] | 26 [23-28] | 0.30^a^ |
| MoCA (median z-score, IQR) | -1.31 [-2.77-0.51] | -0.44 [-1.21-0.47] | 0.29^a^ |
| Episodic memory (median z-score, IQR) | -1.35 [-2.33 to -0.12] * | -0.45  [-1.41-0.34] | 0.13^a^ |
| Working memory  (median z-score, IQR) | -0.04 [-0.87-0.32] ** | -0.37 [-0.82-0.36] | 0.68^a^ |
| Executive function (median z-score, IQR) | -0.19 [-0.60-0.16] * | 0.16  [-0.45-0.60] | 0.16^a^ |
| Processing speed  (median z-score, IQR) | -0.58 [-2.40-0.05] * | -0.63 [-1.53-0.13] | 0.31^a^ |
| Visuospatial^†^ (median z-score, IQR) | -0.70 [-1.32—0.19] | -0.72 [-1.22-0.38] | 0.58^a^ |
| Verbal fluency (median z-score, IQR) | -0.74  [-1.37 to -0.09] * | -0.49 [-1.10-0.18] | 0.31^a^ |
| CI per domain (n (%))  Global cognition | 10 (50%) * | 5 (20%) | 0.096^d^ |
| Episodic memory | 7 (37%) * | 4 (20%) | 0.30^d^ |
| Working memory | 2 (10%) ** | 1 (5%) | 0.46^d^ |
| Executive function | 3 (16%) * | 2 (10%) | 0.66^d^ |
| Processing speed | 8 (40%) | 5 (25%) | 0.50^d^ |
| Visuospatial^†^ | 2 (15%) | 2 (11%) | 1.0^d^ |
| Verbal fluency | 4 (21%) * | 2 (10%) | 0.40^d^ |
| Single domain CI^‡^ | 2 (11%) | 3 (15%) | 1.0^d^ |
| Multidomain CI^‡^ | 8 (42%) | 5 (25%) | 0.32^d^ |

* Data missing from 1 individual, ** Data missing from 2 individuals, see results section above for further details, † BIONIC only; 14 sCAA with AD pathology, 24 sCAA patients without AD pathology; ‡ the visuospatial domain is missing in patients from LUMC, multidomain cognitive impairment is based on one domain less than RUMC. a = Mann-Whitney U test, b = chi-square test, c = Fisher-Freeman-Halton Exact Test, d = Fisher exact test. Percentages are rounded so may not be always add up to 100%. Abbreviations: CAA = sCAA = sporadic cerebral amyloid angiopathy, CMB = cerebral microbleeds, CSS = cortical superficial siderosis, EPVS-CSO = enlarged perivascular spaces in the centrum semiovale, IQR = interquartile range, nHV = normalized hippocampal volume, MoCA=Montreal Cognitive Assessment, SVD = small vessel disease.

**Table S3: Scores on neuropsychiatric questionnaires in participants with sCAA without prior ICH, stratified to CSF profile indicative of AD**

|  | **sCAA-AD+, no ICH (n=23)** | **sCAA-AD-, no ICH (n=19)** | **P-value**  **sCAA with vs without AD** |
| --- | --- | --- | --- |
| Apathy- self reported†  Apathy score (median, IQR)  Apathy, presence (n, %) | 9 [4-17] 5 (39%) | 7 [5-16] 5 (33%) | 0.66^a^ 1.0^b^ |
| Apathy- informant  Apathy score (median, IQR)  Apathy presence (n, %) | 12 [6-22]* 10 (45%)* | 10 [7-14]  5 (26%) | 0.29^a^ 0.33^b^ |
| NPI-Q symptoms   Delusions (n, %) | 1 (4%) | 0 | 1.0^c^ |
| Hallucinations (n, %) | 0 | 0 | - |
| Agitation/aggression (n, %) | 8 (35%) | 2 (11%) | 0.08^c^ |
| Dysphoria/depression (n, %) | 6 (26%) | 2 (11%) | 0.26^c^ |
| Anxiety (n, %) | 4 (17%) | 1 (5%) | 0.36^c^ |
| Euphoria/elation (n, %) | 1 (5%) | 1 (4%) | 1.0^c^ |
| Apathy/indifference (n, %) | 5 (22%) | 4 (21%) | 1.0^c^ |
| Disinhibition (n, %) | 2 (9%) | 2 (11%) | 1.0^c^ |
| Irritability/lability (n, %) | 10 (43%) | 6 (32%) | 0.32^c^ |
| Aberrant motor  behaviours (n, %) | 1 (5%) | 0 | 0.45^c^ |
| Nighttime behavioural   disturbances (n, %) | 2 (10%) | 1 (5%) | 1.0^c^ |
| Appetite/eating  disturbances(n, %) | 2 (9%) | 2 (11%) | 1.0^c^ |
| NPI-Q total score  (median, IQR) | 2 [0-3] | 0 [0-2] | 0.19^a^ |
| NPI-Q no. of symptoms  No symptoms (n, %)  1 or 2 symptoms (n, %)   3 or more symptoms (n, %) | 9 (39%)  6 (21%)  8 (35%) | 12 (63%)  3 (16%)  4 (21%) | 0.39^d^ |

* Data missing from 1 individual, † BIONIC only; 12 sCAA with CSF biomarker status indicative of AD pathology, 15 sCAA patients without CSF biomarker status indicative of AD pathology. a = Mann-Whitney U test, b = chi-square Exact Test, c = Fisher exact test chi-square test, d = Fisher-Freeman-Halton Exact Test. Percentages are rounded so may not be always add up to 100%. Abbreviations: AD = Alzheimer’s disease, CAA = cerebral amyloid angiopathy, CSF = cerebrospinal fluid, NPI-Q = neuropsychiatric inventory questionnaire, IQR = interquartile range

**References**

1. Starkstein SE, Mayberg HS, Preziosi TJ, Andrezejewski P, Leiguarda R, Robinson RG. Reliability, validity, and clinical correlates of apathy in Parkinson's disease. J Neuropsychiatry Clin Neurosci. 1992;4(2):134-9.

2. Cummings JL, Mega M, Gray K, Rosenberg-Thompson S, Carusi DA, Gornbein J. The Neuropsychiatric Inventory: comprehensive assessment of psychopathology in dementia. Neurology. 1994;44(12):2308-14.

3. Nasreddine ZS, Phillips NA, Bédirian V, Charbonneau S, Whitehead V, Collin I, et al. The Montreal Cognitive Assessment, MoCA: a brief screening tool for mild cognitive impairment. Journal of the American Geriatrics Society. 2005;53(4):695-9.

4. Lezak MD. Neuropsychological assessment: Oxford University Press, USA; 2004.

5. Reitan RM, Wolfson D. The halstead-reitan neuropsychological test battery and aging. Clinical Gerontologist: The Journal of Aging and Mental Health. 1986.

6. Meyers JEaM, K.R. . Rey Complex Figure Test and Recognition Trial: Professional Manual. Psychological Assessment Resources O, editor1995.

7. Smith A. Symbol digit modalities test: Western psychological services Los Angeles; 1973.

8. Wechsler D. Wechsler Adult Intelligence Scale-Fourth Edition-Nederlandse Bewerking: Pearson Assesment and Information BV.; 2012.

9. Hammes J. Stroop kleur-woord Test: Dutch manual. Lisse, the Netherlands: Swets & Zeitlinger; 1978.

10. Van Der Elst W, Van Boxtel MP, Van Breukelen GJ, Jolles J. Normative data for the Animal, Profession and Letter M Naming verbal fluency tests for Dutch speaking participants and the effects of age, education, and sex. Journal of the International Neuropsychological Society. 2006;12(1):80-9.
